# Thermoelectric Properties of Flexible PEDOT:PSS/Polypyrrole/Paper Nanocomposite Films

**DOI:** 10.3390/ma10070780

**Published:** 2017-07-11

**Authors:** Jun Li, Yong Du, Runping Jia, Jiayue Xu, Shirley Z. Shen

**Affiliations:** 1 School of Materials Science and Engineering, Shanghai Institute of Technology, 100 Haiquan Road, Shanghai 201418, China; 156081106@mail.sit.edu.cn (J.L.); Jiarp@sit.edu.cn (R.J.); 2 CSIRO Manufacturing, Clayton South, VIC 3169, Australia; shirley.shen@csiro.au

**Keywords:** polypyrrole, paper, composite films, flexible, thermoelectric properties

## Abstract

Flexible poly(3,4-ethylenedioxythiophene):poly(styrenesulfonate)/polypyrrole/paper (PEDOT:PSS/PPy/paper) thermoelectric (TE) nanocomposite films were prepared by a two-step method: first, PPy/paper nanocomposite films were prepared by an in situ chemical polymerization process, and second, PEDOT:PSS/PPy/paper TE composite films were fabricated by coating the as-prepared PPy/paper nanocomposite films using a dimethyl sulfoxide-doped PEDOT:PSS solution. Both the electrical conductivity and the Seebeck coefficient of the PEDOT:PSS/PPy/paper TE nanocomposite films were greatly enhanced from 0.06 S/cm to ~0.365 S/cm, and from 5.44 μV/K to ~16.0 μV/K at ~300 K, respectively, when compared to the PPy/paper TE nanocomposite films. The thermal conductivity of the PEDOT:PSS/PPy/paper composite film (0.1522 Wm^−1^K^−1^ at ~300 K) was, however, only slightly higher than that of the PPy/paper composite film (0.1142 Wm^−1^K^−1^ at ~300 K). As a result, the *ZT* value of the PEDOT:PSS/PPy/paper composite film (~1.85 × 10^−5^ at ~300 K) was significantly enhanced when compared to that of the PPy/paper composite film (~4.73 × 10^−7^ at ~300 K). The as-prepared nanocomposite films have great potential for application in flexible TE devices.

## 1. Introduction

Nowadays, more and more waste heat is generated [1]. Thermoelectric (TE) materials offer a promising solution to convert waste heat into electrical energy. Furthermore, TE devices have many virtues, such as easy maintenance and high reliability, as well as being environmentally friendly [2]. Therefore, TE materials have attracted significant attention in the last few decades. The performance of a TE material is evaluated by the material’s dimensionless figure of merit, *ZT = S^2^σT/κ* (where *T*, *S*, *σ*, and *κ* are the absolute temperature, Seebeck coefficient, electrical conductivity, and thermal conductivity, respectively) [3].

Organic conducting polymers, such as polypyrrole (PPy), poly(3-hexilthiophene) (P3HT), poly(3,4-ethylenedioxythiophene):poly(styrenesulfonate) (PEDOT:PSS), and polyaniline (PANi), have about one order of magnitude lower thermal conductivities (~0.02 Wm^−1^K^−1^–0.542 Wm^−1^K^−1^ [4,5]) than those of inorganic TE materials, such as Bi-Te- and Pb-Te-based alloys. The low thermal conductivity of organic conducting polymers represents a benefit to enhance its TE properties.

PPy, an organic conducting polymer, has great potential to be used in TE materials, because it has relatively high electrical conductivity after being doped with suitable dopants (~340 S/cm at room temperature [6]), and low thermal conductivity (~0.15 Wm^−1^K^−1^ at 310 K [7]). So far, there are mainly three methods for the preparation of PPy, e.g., electrochemical polymerization [8], in situ polymerization [9,10,11], and gas phase polymerization [12]. For example, Lee et al. [13] fabricated PPy films by an electrochemical polymerization method, after which the electrical conductivity and Seebeck coefficient of the PPy films was ~153 S/cm and ~7.14 μV/K at 300 K, respectively. Song et al. [11] prepared PPy nanoparticles by an in situ polymerization method, and the electrical conductivity and Seebeck coefficient of the PPy nanoparticles was ~19.6 S/cm and ~7 μV/K at room temperature, respectively. Different preparation methods have a significant effect on the TE properties of PPy.

Recently, more and more researchers have used paper as a substrate for the preparation of flexible composite materials used for flexible solid-state supercapacitors [14], flexible solar cells [15], and active matrix displays [16]. This is mainly because: (1) paper has many advantages, such as a low price, low density, environmental friendliness, bendability, and flexible, etc. [17]; (2) paper is the cheapest flexible substrate, and it has low thermal conductivity [18], which is also a benefit for TE materials; (3) the thermal stability of paper is much better than polymer substrates such as polyethylene naphthalate or polyethylene terephthalate [19]; and (4) hydrogen bonding forms between the hydroxyl group of paper and the N at PPy rings [14]. For example, Yuan et al. [14] fabricated flexible conductive PPy-coated paper by a soaking and polymerizing process, and assembled flexible solid-state supercapacitors with PPy/paper composite electrodes. Li et al. [20] prepared a cellulose/PPy conductive paper composite by an in situ chemical polymerization method and investigated the surface resistivity stability of the composite. However, to the best of our knowledge, no research on the TE properties of PPy/paper composite materials has been reported. In order to fabricated large-scale flexible TE materials, the choice of a substrate is very important. Since paper has many advantages, as discussed above, we were inspired to fabricate paper-based composite films and explore their applications in the TE area.

PEDOT:PSS is one of the most successful organic conducting polymers; furthermore, its TE properties can be greatly improved by doping with dopants and/or post-treatment [21,22,23,24,25,26,27,28,29]. For example, Kim et al. [21] spin-coated ethylene glycol (EG) or dimethyl sulfoxide (DMSO)-doped PEDOT:PSS films on a glass substrate and then immersed the films in EG solvent to decrease the PSS concentration; as a result, a highest *ZT* value of 0.42 was achieved at room temperature. PEDOT:PSS aqueous solution (PH1000) has been commercially produced on a large scale by H.C. Starck Clevios company [30]. Many researchers have used this conducting solution to prepare TE composites and TE power generators [19,31]. For example, Jiang et al. [18] fabricated free-standing PEDOT:PSS/paper composite films by directly writing PEDOT:PSS solution on a paper substrate. The electrical conductivity, Seebeck coefficient, and thermal conductivity of the PEDOT:PSS/paper composite film was 0.2 S/cm, 30.6 μV/K, and ~0.156 Wm^−1^K^−1^ at 300 K, respectively. This work shows that the use of paper as a substrate can decrease the thermal conductivity of PEDOT:PSS (0.22 Wm^−1^K^−1^–0.36 Wm^−1^K^−1^ [24,32]). Wei et al. [19] screen-printed PEDOT:PSS TE modules on a paper substrate and reported the power output of the TE modules.

Most recently, Lay et al. [30] prepared a cellulose nanofiber suspension and then mixed it with PEDOT:PSS solution to form a cellulose nanofiber-PEDOT:PSS suspension, after which pyrrole monomer and FeCl_3_ solution were added into the above suspension, and the cellulose nanofiber-PEDOT:PSS-PPy nanopapers were prepared after being filtered and dried. The result shows that there are hydrogen-bonding interactions between the PEDOT:PSS and the hydroxyl functionalized cellulose nanofiber. A synergistic phenomenon between PPy and PEDOT:PSS on cellulose nanofiber enhances the electrical conductivity of the cellulose nanofiber-PEDOT:PSS-PPy nanopapers. However, no research on the TE properties of PEDOT:PSS/PPy/paper composite materials has been reported.

In this work, flexible PPy/paper TE composite films were prepared by an in situ chemical polymerization method, and then coated by PEDOT:PSS to further enhance the electrical conductivity of the PPy/paper TE composite films. The influence of PEDOT:PSS coating on the morphology and TE properties of PPy/paper composite films is investigated. To the best of our knowledge, this is the first time that the TE properties of flexible PEDOT:PSS/PPy/paper composite films are reported.

## 2. Materials and Methods

### 2.1. Materials

Pyrrole monomer and dimethyl sulfoxide (DMSO) were purchased from Sigma-Aldrich (Shanghai, China). FeCl_3_·6H_2_O and absolute ethanol were purchased from Sinopharm Chemical Reagent Co., Ltd. (Shanghai, China). PEDOT:PSS (Clevios™ PH 1000) was obtained from H. C. Stark, Inc. (Hanau, Germany). Paper, a common printing paper, was purchased from Zhanjiang Chenming Paper Co., Ltd. (Zhanjiang, China, the density and thickness of which was 70 g/m^2^ and 96 μm, respectively). All the materials were used without further treatment or purification.

### 2.2. Preparation of Flexible PEDOT:PSS/PPy/Paper Composite Films

**Figure 1:**
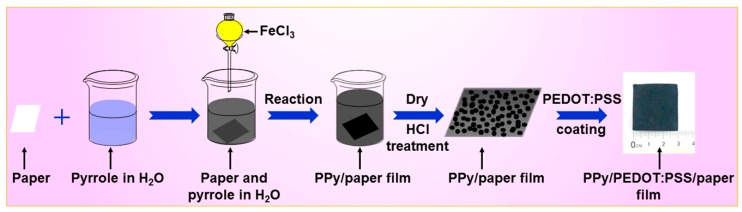
The fabrication process of the PPy/paper and PEDOT:PSS/PPy/paper composite films.

For the preparation method, 0.55 mL of pyrrole monomer was dissolved in 100 mL of deionized water (Solution I, the volume content of pyrrole monomer was 0.547%). A piece of the paper (3 × 3 cm) was soaked in Solution I, which was continuously stirred at 50 rpm for 30 min. The pyrrole monomer was absorbed onto the surface and even diffused into the bulk of paper. Then, 8.65 g of FeCl_3_·6H_2_O as an oxidant was dissolved in 100 mL of deionized water (Solution II), which was added to Solution I to initiate the polymerization. The solution was constantly stirred at 50 rpm for 8 h at room temperature (RT) to form PPy nanoparticles on the surfaces of paper. The PPy/paper composite film was then taken out and washed with deionized water and absolute ethanol successively three times. The washed PPy/paper composite film was dried at 60 °C for 12 h. The dried PPy/paper composite film was soaked in 1 mol/L HCl for 12 h and then dried again at 60 °C for 12 h. An appropriate amount of DMSO was mixed with PEDOT:PSS solution to form a mixture of 5 wt % of DMSO/PEDOT:PSS. The mixture was sonicated for 1 h at RT. The PPy/paper composite film was immersed in the DMSO/PEDOT:PSS solution for 30 min, and then dried at 130 °C for 15 min to form PEDOT:PSS/PPy/paper composite film. Figure 1 shows the fabrication process of the PPy/paper and PEDOT:PSS/PPy/paper composite films.

### 2.3. Characterizations

The composition and morphology of the samples were characterized by X-ray photoelectron spectroscopic (XPS) (PHI 5000 VersaProbe (ULVAC-PHI, Chigasaki, Japan) and scanning electron microscopy (SEM; Philips XL 30 FEG, Eindhoven, The Netherlands), respectively. The electrical conductivities and Seebeck coefficients of the samples were measured simultaneously (in a vacuum atmosphere from 300 K to 370 K) on a MRS-3L thin-film thermoelectric test system (Wuhan Giant Instrument Technology Co., Ltd, Wuhan, China). The thermal conductivities of the samples were measured by a transient hot-wire method at room temperature (TC3000E thermal conductivity meter, Xiatech Electronics Co., Ltd., Xi’an, China).

## 3. Results and Discussion

**Figure 2:**
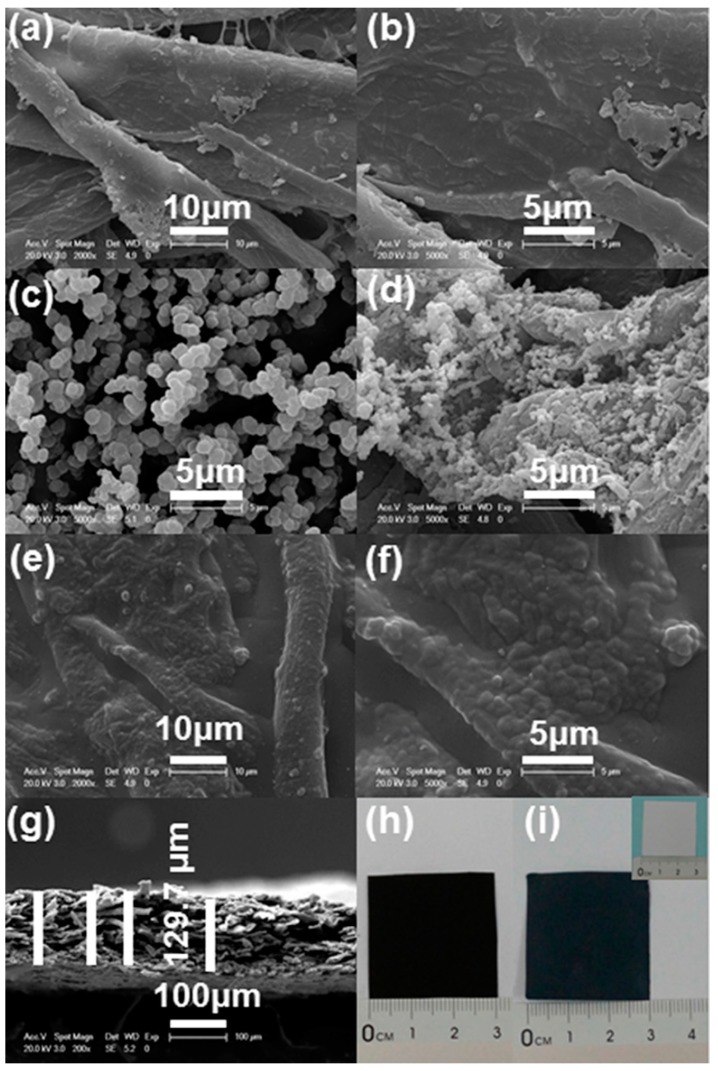
(**a**,**b**) SEM images of paper; (**c**) PPy particles; (**d**) PPy/paper composite film; (**e**,**f**) PEDOT:PSS/PPy/paper nanocomposite film; (**g**) cross-section of PEDOT:PSS/PPy/paper composite film. Photos of (**h**) PPy/paper and (**i**) PEDOT:PSS/PPy/paper composite films. The inset in panel (**i**) is a photo of the common printing paper before any treatment.

Figure 2a–f show the SEM images of surface of the paper, PPy, PPy/paper, and PEDOT:PSS/PPy/paper composite, respectively. The paper consists of microfibers, and the PPy particles were well dispersed on the surface of paper. After PEDOT:PSS is coated, the surfaces of PPy/paper films become smoother. Figure 2g shows an SEM image of a cross-section of the PEDOT:PSS/PPy/paper composite film. The film thickness, measured in Figure 2g to be about 129.7 μm, is a lot thicker than that of the paper (~96 μm). This is mainly because the paper is coated by PPy particles and PEDOT:PSS. Figure 2h,i show the photos of paper, PPy/paper and PEDOT:PSS/PPy/paper composite films, respectively. After the successive polymerization of PPy and coating treatment of PEDOT, the color of the paper changes from white to black.

**Figure 3:**
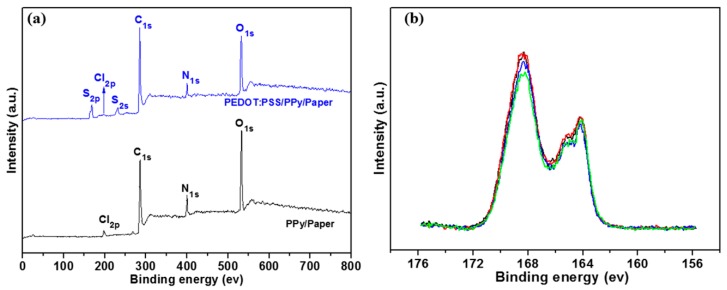
(**a**) XPS (X-ray photoelectron spectroscopy) survey spectra; (**b**) XPS spectra of S2p of the PEDOT:PSS/PPy/paper composite films measured at four different positions in one sample.

The XPS results of PPy/paper and PEDOT:PSS/PPy/paper composite films are shown in Figure 3. The PPy/paper composite film mainly contains C, N, O, and Cl. After PEDOT:PSS treatment, S appears (Figure 3a). The binding energies at around 164.2 eV and 168.4 eV are attributed to the S2p band in PEDOT and S2p band in PSS, respectively (Figure 3b) [33]. Note that the Cl detected is mainly because the samples were treated in HCl. The XPS spectra of S2p of the PEDOT:PSS/PPy/paper composite films measured at four different positions in one sample are very similar (Figure 3b), which indicates that PEDOT:PSS is evenly covered on the surface of the PPy/paper composite film.

**Figure 4:**
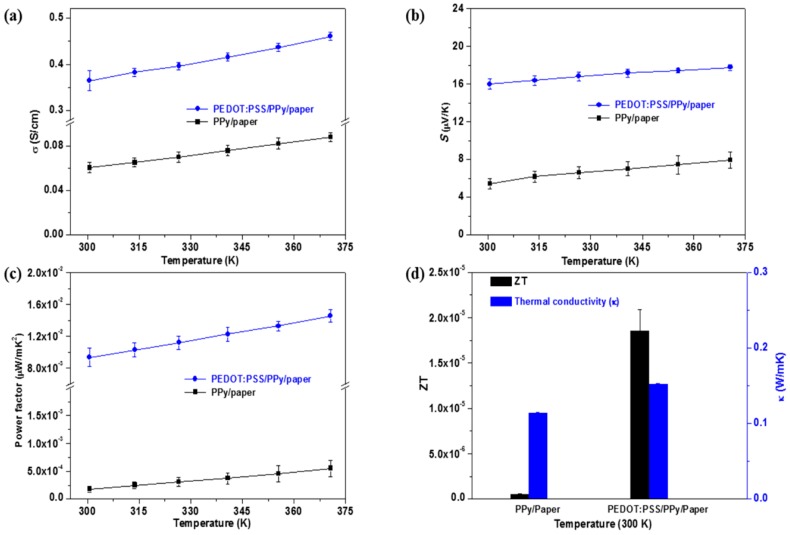
(**a**) Electrical conductivity; (**b**) Seebeck coefficient; (**c**) power factor; and (**d**) *ZT* of the PPy/paper nanocomposite films and the PEDOT:PSS/PPy/paper nanocomposite films.

The electrical conductivity, Seebeck coefficient, power factor (*S^2^σ*), and *ZT* value of the PPy/paper and PEDOT:PSS/PPy/paper composite films are shown in Figure 4. Both the electrical conductivity of PPy/paper and PEDOT:PSS/PPy/paper composite films increase as the temperature increases from 300 K to 370 K (Figure 4a). The electrical conductivity of PEDOT:PSS/PPy/paper composite films is very stable for three different measurements, and it is much higher than that of PPy/paper composite films at the same temperature. This phenomenon can be attributed to the following two reasons: (1) PEDOT:PSS has a much higher electrical conductivity (~753.8 S/cm [31]) than the PPy at RT; (2) After the PEDOT:PSS coating is added, the surface of the PPy/paper composite film becomes smoother, mainly because the PEDOT:PSS was filled in between the PPy and provides a conductive path, which benefits carrier conduction and agrees with the results reported in Reference [31]. Thus, the uniform distribution of PPy and PEDOT:PSS enhances the TE properties of the PEDOT:PSS/polypyrrole/paper nanocomposite films.

The Seebeck coefficient of PPy/paper and PEDOT:PSS/PPy/paper composite films are both positive, manifesting p-type conduction. The Seebeck coefficient of both films also increase with the increase in temperature from 300 K to 370 K. The Seebeck coefficient value of the PPy/paper composite films is 5.4–7.9 uV/K, which is much lower that of PEDOT:PSS/PPy/paper composite films (~16.0–17.8 uV/K) in the temperature range from 300 K to 370 K (Figure 4b).

The power factor of the PEDOT:PSS/PPy/paper composite film is much higher than that of the PPy/paper composite film in the temperature range from 300 K to 370 K (Figure 4c). This is mainly because of the simultaneously increased electrical conductivity and Seebeck coefficient after the PEDOT:PSS coating treatment. A maximum power factor of ~1.5 × 10^−2^ uWm^−1^K^−2^ at 370 K (average value for three measurements) was obtained for the PEDOT:PSS/PPy/paper composite films. It is much higher than that of the PPy/paper composite films (~5.54 × 10^−4^ uWm^−1^K^−2^ at 370 K). However, this value is still much lower than that of the DMSO-doped PEDOT:PSS film at RT (~7.28 uWm^−1^K^−2^ [31]). This may be due to the electrical isolation characteristic of paper.

**Figure 5:**
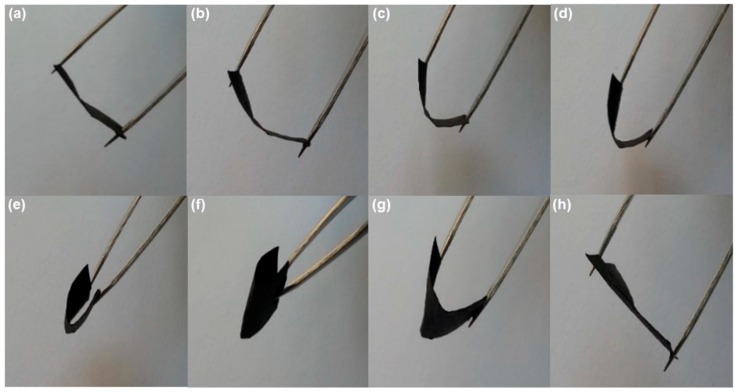
A series of photographs to illustrate the flexibility of the PEDOT:PSS/PPy/paper film. (**a**) Start to bend; (**b**–**e**) bending progress; (**f**) bended state; (**g**) releasing the bend; (**h**) returned to non-bended state as beginning.

Both the electrical conductivity and Seebeck coefficient of PEDOT:PSS/PPy/paper composite films are much higher than those of PPy/paper composite films, while the thermal conductivity of the PEDOT:PSS/PPy/paper composite films (0.1522 Wm^−1^K^−1^ at ~300 K) is only slightly higher than that of PPy/paper composite film (0.1142 Wm^−1^K^−1^ at ~300 K). The *ZT* value of the PEDOT:PSS/PPy/paper composite film (~1.85 × 10−5, at ~300 K) is ~39 times higher than that of the PPy/paper composite film (~4.73 × 10−7, at ~300 K). It is an effective method to enhance the TE properties of PPy/paper composite films by PEDOT:PSS coating, and the coating also has good stability on the paper substrate. Furthermore, the as-prepared PEDOT:PSS/PPy/paper composite films are very flexible; they can be bent easily (Figure 5) and cut into almost any shape. Both sides have the same TE properties. The films thus have potential applications in flexible TE devices, flexible electronics, flexible solid-state supercapacitors, flexible energy storage devices, etc.

## 4. Conclusions

Flexible PEDOT:PSS/PPy/paper thermoelectric composite films were prepared by a two-step method. Compared to PPy/paper TE nanocomposite films, both the electrical conductivity and Seebeck coefficient of the PEDOT:PSS/PPy/paper TE nanocomposite films were greatly enhanced, while the thermal conductivity of the PEDOT:PSS/PPy/paper composite films is only slightly higher than that of PPy/paper composite films. The *ZT* value of the PEDOT:PSS/PPy/paper composite film is, therefore, about 39 times higher than that of the PPy/paper composite films. The as-prepared nanocomposite films have great potential for application in flexible, wearable TE devices. Finally, n-type paper-based TE nanocomposite materials, after doping with suitable dopants, and a real TE couple for TE generation will be investigated in the future to facilitate the investigation into the above potential applications.
